# Investigating the Effects of Chiropractic Spinal Manipulation on EEG in Stroke Patients

**DOI:** 10.3390/brainsci10050253

**Published:** 2020-04-27

**Authors:** Muhammad Samran Navid, Imran Khan Niazi, Dina Lelic, Rasmus Bach Nedergaard, Kelly Holt, Imran Amjad, Asbjørn Mohr Drewes, Heidi Haavik

**Affiliations:** 1 Mech-Sense, Department of Gastroenterology and Hepatology, Aalborg University Hospital, 9000 Aalborg, Denmark; m.navid@rn.dk (M.S.N.); dilelic@gmail.com (D.L.); r.nedergaard@rn.dk (R.B.N.); amd@rn.dk (A.M.D.); 2 Department of Clinical Medicine, Aalborg University, 9000 Aalborg, Denmark; 3 Centre for Chiropractic Research, New Zealand College of Chiropractic, Auckland 1060, New Zealand; kelly.holt@nzchiro.co.nz (K.H.); imran.amjad@nzchiro.co.nz (I.A.); Heidi.Haavik@nzchiro.co.nz (H.H.); 4 Faculty of Health & Environmental Sciences, Health & Rehabilitation Research Institute, AUT University, Auckland 0627, New Zealand; 5 Centre for Sensory-Motor Interactions, Department of Health Science and Technology, Aalborg University, 9220 Aalborg, Denmark; 6 Riphah College of Rehabilitation Sciences, Riphah International University, Islamabad 46000, Pakistan

**Keywords:** chiropractic, stroke, electroencephalography, somatosensory evoked potentials, brain waves, spinal manipulation

## Abstract

**Objective:** The purpose of this study was to evaluate the impact of chiropractic spinal manipulation on the early somatosensory evoked potentials (SEPs) and resting-state electroencephalography (EEG) recorded from chronic stroke patients. **Methods:** Seventeen male patients (53 ± 12 years old) participated in this randomized cross-over study. The patients received chiropractic spinal manipulation and control intervention, in random order, separated by at least 24 hours. EEG was recorded before and after each intervention during rest and stimulation of the non-paretic median nerve. For resting-state EEG, the delta-alpha ratio, brain-symmetry index, and power-spectra were calculated. For SEPs, the amplitudes and latencies of N20 and N30 peaks were assessed. Source localization was performed on the power-spectra of resting-state EEG and the N30 SEP peak. **Results:** Following spinal manipulation, the N30 amplitude increased by 39%, which was a significant increase compared to the control intervention (*p* < 0.01). The latency and changes to the strength of the cortical sources underlying the N30 peak were not significant. The N20 peak, the resting-state power-spectra, delta-alpha ratio, brain-symmetry index, and resting-state source localization showed no significant changes after either intervention. **Conclusion:** A single session of chiropractic spinal manipulation increased the amplitude of the N30 SEP peak in a group of chronic stroke patients, which may reflect changes to early sensorimotor function. More research is required to investigate the long-term effects of chiropractic spinal manipulation, to better understand what impact it may have on the neurological function of stroke survivors.

## 1. Introduction

Stroke is the second-most common cause of death globally, preceded only by ischemic heart disease [1]. It has a high prevalence, affecting approximately 200 persons per 100,000 [2] and requires extensive rehabilitation, with high economic and social costs (~€21,000 per patient in 2010) [3]. Upper limb function is often affected by stroke, and its improvement has been identified as one of the top ten research priorities by stroke survivors, caregivers, and clinicians [4].

The predictors known to identify inefficient upper limb sensorimotor recovery are increased stroke severity, more severe early somatosensory and motor impairments, and the presence of visuospatial neglect [5]. Somatosensory evoked potentials (SEPs) are frequently used as a measure of somatosensory processing after stroke, and abnormalities in the median nerve SEPs often predict functional outcomes for stroke survivors [6,7,8,9,10,11,12]. Compared to healthy controls or the same subjects’ unaffected hemisphere, typical median nerve SEP abnormalities are found in affected hemispheres of stroke survivors. These are abnormally long interpeak intervals (such as N9-N13, N13-N20), absent or severely reduced SEP peak amplitudes (such as N20 and P22 amplitudes), and prolonged latency of some SEP peaks (such as 2-5 standard deviations higher latency of N20) [7,10,11,13,14]. A recent systematic review suggested that acute, inter-hemispheric imbalances involving higher alpha event-related synchronization in the affected hemisphere could be more pronounced in those with moderate upper limb motor impairments [11]. 

The cortical and subcortical areas associated with the movement are interconnected [15], and the active networks can be seen during the assessment of the electrical resting-state activity of the brain. After a stroke, the reorganization of these electrophysiological networks can result in changes, for example, in the functional connectivity [16], and the power spectrum [17]. The changes in resting activity have been associated with motor dysfunction during movements [18]. Increased activity of the slower oscillations, i.e., increased power of the delta and theta frequency bands have been found to be related to brain damage due to, for example, stroke, brain hemorrhage, tumors and traumatic injury [19,20,21,22]. Finnigan et al. [23] suggested that the delta-alpha ratio (DAR) and pairwise-derived brain symmetry index (BSI) calculated from the resting-state electroencephalography (EEG) can be important predictors of neurological function in stroke survivors. Increased DAR and BSI were found in the (sub-) acute stage after stroke [23,24,25]. However, only BSI in lower frequency bands was increased in the chronic stroke survivors [17].

The possible mechanisms involved in post-stroke motor recovery consist of facilitation and modulation of neural plastic changes in the brain [26]. In the past two decades, many studies have shown that chiropractic spinal manipulation has a neural plastic effect on the central nervous system (CNS) [27,28,29]. Numerous studies have demonstrated altered central processing, such as those associated with somatosensory processing, sensorimotor integration, motor control, and pain after (usually a single session of) chiropractic spinal manipulation, which suggests that the chiropractic intervention can rapidly affect central neural function in a variety of ways [27,28,30,31,32,33,34,35,36,37,38]. Several of these studies have shown that chiropractic care can alter the amplitudes of several SEP peaks, in particular, the N20 and N30 peaks [32,36,39,40]. The most consistent change following chiropractic care is a reduction in amplitude of the N30 SEP peak [32,36,39,40], which has been shown to occur primarily in the prefrontal cortex [39]. The prefrontal cortex is highly involved in sensorimotor integration and motor control [32,34,35,38]. Thus, the changes in the prefrontal cortex might be the reason why a single session of chiropractic care could impact and improve force development, as has recently been shown in a chronic stroke population [41]. However, the effects of chiropractic care on post-stroke sensory and motor recovery have not yet been adequately studied.

Considering that N30 SEP peak amplitudes have been shown to decrease due to reduced cerebral blood flow [42], and previous studies have consistently shown a decrease in N30 SEP peak amplitude after chiropractic care [32,36,39,40] albeit, in a different population, we considered it to be worth investigating what effect chiropractic care has on N30 SEP peaks, as well as other measures of brain activity, in stroke survivors. Therefore, this study aimed to evaluate the effects of a single session of chiropractic spinal manipulation on the resting-state EEG, early somatosensory evoked potentials, and the strength of its corresponding brain sources in stroke survivors.

## 2. Methods

The study used a randomized controlled cross-over design and was conducted at Railway General Hospital in Rawalpindi, Pakistan. The Riphah International University Research Ethics Committee, Pakistan, approved the study (ref # Riphah/RCRS/REC/000118). The study was also approved by the New Zealand College of Chiropractic Research Review Committee. The study was conducted in accordance with the Declaration of Helsinki.

### 2.1. Subjects

Nineteen stroke patients (all males, 53 ± 12 years old) participated in this study. By using a purposive sampling technique, the subjects were recruited from the outpatient facility of the rehabilitation department, where they came for their conventional physical rehabilitation. The subjects gave their written informed consent to participate in the study. The patient details are given in Table 1. Although it was not part of the inclusion criteria, all subjects recruited were males and naïve to chiropractic care. 

Before enrolling in the study, the subjects were introduced to the lab environment. Subjects were eligible to participate if they had suffered from a stroke at least 12 weeks before their involvement in the study and had a Fugl-Meyer Assessment score (combined upper and lower limb) of less than or equal to 85 (i.e., they had significant motor impairment [43]). The subjects were ineligible to participate if they showed no evidence of spinal dysfunction (i.e., presence of vertebral subluxation indicators identified by a chiropractor), had absolute contraindications to spinal adjustments (including spinal fracture, atlantoaxial instability, spinal infection, spinal tumor, or cauda equina syndrome), or previously had a significant adverse response to chiropractic care.

### 2.2. Experimental Protocol

The subjects participated in two sessions, chiropractic and control, in random order, separated by at least 24 hours. The balanced randomization scheme was generated using Minimizer (Microsoft Corp. Redmond, WA, USA). The subjects were not informed beforehand that one of the interventions would be a control intervention.

Each session consisted of recording resting-state EEG, followed by SEPs evoked by electrical stimulation of the median nerve of the non-paretic limb. Both recordings were done before and after the intervention. During each session, the subjects were seated comfortably in a chair, in front of a screen and were asked to keep their eyes open and be relaxed to reduce the contamination of EEG signals.

The study was single-blinded; therefore, the subjects did not know which intervention they received. The data analysis was performed by blinding the analyst through assigning random numbers to each dataset (recording) of every subject.

### 2.3. Interventions

The chiropractic spinal manipulation and control interventions were similar to those used in previous studies [27,28,37,39,41] that have investigated the neurophysiological effects of chiropractic spinal adjustments. The same chiropractor performed the actual and control adjustments. At the end of the second session, the subjects were asked if they felt that they had undergone active treatment in each session (‘yes’ or ‘no’).

#### 2.3.1. Chiropractic Manipulation

The standard adjustment techniques used by the chiropractors, also known as spinal manipulation, were used in the chiropractic spinal adjustment session. The chiropractor performed manual high-velocity low-amplitude adjustments or instrument-assisted adjustments to the spine or pelvic joints [44]. The chiropractor used standard clinical indicators of spinal and pelvic dysfunction to decide where to adjust. [45] These indicators included tenderness to palpation, restricted intersegmental motion, muscle asymmetry, and blocked jopint play or end-feel. Chiropractic adjustments were applied to multiple spinal segments if required. 

#### 2.3.2. Control Manipulation

The control intervention was performed by the same chiropractor who provided the chiropractic intervention. In the control session, the chiropractor interacted with the patient in a similar way to the active session, including assessing the spine and pelvis for dysfunction and then moving and setting up the patient as if they were going to apply an adjustive thrust. However, during the adjustment set up, the chiropractor took care not to provide an adjustive thrust or to take a vertebral segment that was deemed to be subluxated to tension. The control session was designed to control for the interaction and time taken during the chiropractic intervention and to control for the mechanoreceptive input associated with the chiropractor assessing the patient’s spine, while ensuring the afferent input associated with the adjustive thrust was minimized.

### 2.4. Median Nerve Stimulation

The median nerve was stimulated using electrical pulses delivered by the electrical stimulator (Digitimer DS7AH, Hertfordshire, UK) to evoke SEPs. The stimulation electrodes (Neuroline 700, AMBU A/S, Ballerup, Denmark) were placed at the wrist. The motor threshold was defined as the lowest current intensity, which elicited a visible twitch of the thumb. Before and after each intervention, a total of 1000 electrical pulses were given to the median nerve of the non-paretic limb. The stimulation pulse was monophasic, with a width of 0.2 ms and a frequency of 2.3 Hz.

### 2.5. EEG

The EEG was recorded at a sampling rate of 2048 Hz from 62 channels using a REFA amplifier (TMSi, Twente, The Netherlands) according to the 10-20 electrode system. The ground electrode was placed at AFz. The impedance of the electrodes was kept below 10 kΩ. The subjects were asked to focus on a white fixation cross with black background displayed in the center of a computer screen while minimizing eye blinks, eye movements, and facial movements.

The EEG was analyzed offline using EEGLAB version 14.1.1 [46], ERPLAB version 6.1.4 [47], and FieldTrip version 20180912 [48] running on MATLAB 2015b (The MathWorks, Inc., Natick, MA, USA). We developed custom scripts in MATLAB utilizing EEGLAB, ERPLAB, FieldTrip, and MATLAB functions to perform the analysis. The analysis pipeline has been previously described in detail [49].

The raw EEG was truncated to keep up to an additional 30 s of data in the beginning and end of the recordings to reduce filter artifacts. The PREP pipeline [50] was used to identify bad channels, remove line noise, and obtain the average referenced data.

#### 2.5.1. Resting-State EEG

The resting-state EEG was recorded for 5 minutes. For artifact detection and correction, the PREPed EEG was high-pass filtered using a Kaiser windowed FIR filter (β = 5.653) with a cutoff frequency of 1 Hz and an order of 4948 equivalent to the transition bandwidth of 1.5 Hz. After cleaning the data, the channels of the subjects with the right affected hemisphere were mirrored in the sagittal plane to model all subjects having the lesion(s) in the left hemisphere. The data from subjects with at least 2 min of clean EEG per session were used for further analysis. The analyses were performed on the data downsampled to 256 Hz to reduce the computational time and remove redundant frequency spectrum not required in the analysis. The EEG was segmented into 2 s long epochs to encompass two cycles of the lowest frequency of interest (1 Hz). For any session with clean data longer than 2 min, 60 epochs were randomly selected to equalize the length of data across sessions and subjects.

##### Spatio-Spectral Power

The power spectra were calculated between 1 and 80 Hz using the Fourier basis with a 2 s wide Hanning window. Afterwards, the average power of each classical frequency band (delta (1–4 Hz), theta (4.1–8 Hz), alpha (8.1–12 Hz), beta (12.1–32 Hz), and gamma (32.1–80 Hz)) was computed.

##### DAR

For each channel c, the DAR was calculated to get the ratio of the mean delta power to the mean alpha power as

(1) $$DAR_{c}=\frac{{\langle P_{c}\left(f\right)\rangle }_{f=1,\;\ldots ,\;4\;Hz}}{{\langle P_{c}\left(f\right)\rangle }_{f=8,\;\ldots ,\;\;12\;Hz}}$$

The global DAR was calculated by averaging the ratios across all N EEG channels:

(2) $$DAR=\frac{1}{N}\overset{N}{\underset{c=1}{\sum }}DAR_{c}$$

##### BSI

The pairwise-derived BSI was calculated by taking the absolute difference of the mean spectral power between the homologous left cL and right cR EEG channels. The BSI was calculated over the 1–25 Hz range [17] by

(3) $$BSI_{cp}={\langle |\frac{P_{cR}\left(f\right)-P_{cL}\left(f\right)}{P_{cR}\left(f\right)+P_{cL}\left(f\right)}|\rangle }_{f=1,\ldots ,25\;Hz}$$

The BSI across all channel pairs cp was averaged to get global BSI:

(4) $$BSI=\frac{2}{N}\overset{N/2}{\underset{cp=1}{\sum }}BSI_{cp}$$

The range of BSI is 0 to 1, where 0 means perfect symmetry, and 1 represents no symmetry between channel pairs.

Since BSI does not take the direction of asymmetry into account, the direction of asymmetry was calculated by not taking the absolute of Equation (3):

(5) $$BSI_{dir_{cp}}={\langle \frac{P_{cR}\left(f\right)-P_{cL}\left(f\right)}{P_{cR}\left(f\right)+P_{cL}\left(f\right)}\rangle }_{f=1,\ldots ,25\;Hz}$$

The values were averaged over all channel pairs:

(6) $$BSI_{dir}=\frac{2}{N}\overset{N/2}{\underset{cp=1}{\sum }}BSI_{dir_{cp}}$$

$BSI_{dir}$ ranges from −1 to +1, where 0 means perfect symmetry, positive values mean higher power in the right hemisphere compared to the left hemisphere and vice versa for the negative values. $BSI_{dir}$ for all subjects was multiplied by −1. This way, the positive values characterized higher power in the affected hemisphere compared to the unaffected hemisphere, and vice versa for the negative values [17].

In addition to BSI for the 1–25 Hz band, separate BSI calculations were made for the delta, theta, alpha, beta, and gamma bands. The mid-line channels were not included in the BSI calculation.

##### Source Localization

The localization of electrical activity in the brain during rest was estimated in the LORETA-KEY software, version 20151222 [51] (freely available at www.uzh.ch/keyinst/loreta). For source localization, we used sLORETA, which is a linear inverse algorithm that estimates the distribution of cortical generators of the EEG in three-dimensions, with lowest localization error compared to several other linear inverse methods [51]. The sLORETA implementation uses a reference brain from the Montreal Neurological Institute (average MRI brain map (MNI-152)) [52] with cortical gray matter divided into 6239 voxels with a resolution of 5 mm.

For sLORETA analysis, the EEG was segmented into 8 s long epochs to obtain smooth power spectral density. The data from subjects with at least 2 minutes of clean EEG per session were used. For any session with clean data longer than 2 minutes, 15 epochs were randomly selected to equalize the length of data across sessions and subjects. The sLORETA was performed in the frequency domain where cross-spectral matrices for each subject were computed in the LORETA-KEY software for the same five frequency bands as in the power spectral analysis above. Subsequently, the cross-spectral matrices were averaged for each subject and used as input for the sLORETA.

#### 2.5.2. SEPs

The EEG recorded during the median nerve stimulation of the non-paretic limb was analyzed. For artifact detection and correction, the PREPed EEG was high-pass filtered using a 2nd order Butterworth filter with a cutoff frequency of 1 Hz. The epochs were extracted from 100 ms before the stimulus to 150 ms after the stimulus and baseline corrected using the pre-stimulus period. After cleaning the data, the channels of the subjects with the right affected hemisphere were mirrored in the sagittal plane to model all subjects having a lesion(s) in the left hemisphere. For each subject, the number of epochs in each session was equalized to the session with a minimum number of epochs (for that subject), by randomly removing clean excess epochs. Finally, the epochs were averaged.

##### SEP Peaks

In this study, we analyzed the amplitudes and latencies of the N20 and N30 SEP peaks. The N20 amplitude was calculated from the central electrode. The most positive (P14) and the most negative (N20) peaks with respect to the stimulus were identified in the time window of 13–20 ms and 20–30 ms, respectively. The N30 amplitude was calculated from the frontal electrode, as done previously [39]. The most positive (P22) and the most negative (N30) peaks with respect to the stimulus were identified in the time window of 15–25 ms and 25–45 ms, respectively. Afterwards, manual inspection was performed, and the identified peaks were verified by an expert in SEP analysis. The N20 and N30 amplitudes were defined as the absolute difference in the amplitudes of these peaks.

##### Source Localization

Brain source modeling was performed in the 18 to 60 ms post-stimulus period using Brain Electrical Source Analysis (BESA) (BESA Research 5.3; MEGIS Software GmbH, Gräfelfing, Germany) software. First, the potential distributions over the scalp from preset voltage dipoles within the brain were calculated. Afterwards, the agreement between the recorded and the calculated field potentials was evaluated. For further analysis, the percentage of data that could not be explained by the model, termed as residual variance (RV), was required to be less than 10%. A 4-shell ellipsoidal model with a radius of 85 mm was used.

The models were first created on the pre-session grand-averages to get an indication of the location and number of sources. Afterwards, LORETA was used on individual pre-sessions to guide the estimation of the location and number of sources. LORETA is a current density model that produces blurred source images, requires no prior constraints, and has high accuracy [53]. Once the dipoles were placed, the model fit was obtained by fixing their locations but allowing their orientations to move freely. The model was then applied to the associated post-sessions. The source activation waveforms were exported to MATLAB 2015b (The MathWorks, Inc., Natick, MA, USA), and brain source strengths for the N30 peak were computed by calculating the area under the curve (AUC) in the 20 to 60 ms post-stimulus period.

#### 2.5.3. Artifact Removal

The same parameters were used for marking epochs as bad for both the resting-state EEG and SEPs. The resting-state EEG was segmented into 0.5 s long epochs. An epoch was considered bad if, for any channel, (i) the amplitude exceeded 100 μV, (ii) peak-to-peak amplitude was more than 150 μV in any sliding window of 200 ms width with a step size of 100 ms, (iii) the amplitude was greater than 100 μV in a step-function with a sliding window 200 ms wide with a step size of 50 ms, (iv) sample-to-sample difference was more than 50 μV, or (v) the amplitude was less than 2 μV for 125 ms (SEPs) or 150 ms (resting-state EEG) (i.e., flat-lined data). For SEPs, the above conditions were not applied to −2 ms to 2 ms period corresponding to the stimulus artifact. Afterwards, all epochs were manually verified to classify into good and bad epochs. The epochs with step-like artifacts in the frontal channels were not removed as they were related to eye-blinks and eye-movements [54].

The high-pass filtered EEG was downsampled to 512 Hz and epoched in a similar way as described above. The bad epochs and bad channels (from PREP) were removed from the data, and adaptive mixture independent component analysis was employed to decompose EEG into maximally independent components i.e., which are spatially fixed and temporally discrete [55]. This algorithm was used since its performance is better than that of other ICA algorithms [56]. The ICA weights obtained were applied to band-pass (0.5–1000 Hz) filtered PREPed data with bad epochs removed. For resting-state EEG, a Kaiser windowed FIR filter (β = 5.653) with an order of 7420 corresponding to the transition bandwidth of 1 Hz was used. For SEPs, a 2nd order Butterworth filter was employed. Afterwards, all the independent components (ICs) were manually categorized into the brain or non-brain components resulting from activities of muscle or eye, or noise of channel or mains. The ICs were categorized based on their spatial distribution (scalp topography), time course, spectrograms, event-related potential (ERP) images, and current dipole models using recommendations from [57,58] and the website https://labeling.ucsd.edu/. The bad ICs were removed, followed by interpolation of the noisy channels to give cleaned datasets.

### 2.6. Statistics

The data are reported as mean ± SD unless otherwise indicated. The statistical significance threshold was set at *p* < 0.05.

Two-way, repeated-measures ANOVAs were used with factors of intervention (Control and Chiropractic) and session (pre and post) to identify the effects of the intervention on the SEPs’ amplitudes and latencies, brain source strengths, DAR and BSI. Tukey’s HSD was used to perform pairwise comparisons. The statistical procedure was performed in MATLAB 2015b (The MathWorks, Inc., Natick, MA, USA).

Non-parametric cluster-based permutation tests [59] were used with a cluster-threshold of 0.05 to evaluate the differences between the interventions based on the global power spectrum of the resting-state. The clusters were defined as two or more channel-frequency pairs, each with *p* < 0.05 from the paired t-test (two-tailed). The maximum of cluster-level statistics, obtained by adding the *t*-values within each cluster, was used as the test statistic. A cluster was considered significant if its Monte Carlo probability for each tail exceeded the threshold of 0.025 compared to the reference distribution, which was approximated by the Monte Carlo method with 5000 permutations.

The statistical procedure for source localization of resting-state EEG was performed in the LORETA-KEY software using non-parametric mapping [60], which utilized Fisher’s random permutation test with 5000 randomizations to control for multiple comparison problem. The paired two-tailed *t*-test was used to find differences in the current sources across the different frequency bands. The tests were used to identify differences between the pre-sessions; the post- and pre-chiropractic sessions; and the post- and pre-control sessions.

## 3. Results

Out of nineteen chronic stroke survivors recruited, two were excluded as they were unable to complete the experiment—one was uncomfortable with the median nerve stimulation, and the other could not follow the instructions of the experimenter. Hence, the analyses were performed on the remaining 17 subjects (53 ± 12 years old). From the questions to evaluate the success of subject blinding, out of the 17 subjects, only two felt that one of the sessions was not an active session, and one of these was correct in the identification of the order of the interventions (chiropractic or control) he received.

### 3.1. Resting-State EEG

The analyses were performed on 16 subjects who had a minimum of 2 minutes of clean resting-state EEG in every session.

#### 3.1.1. Spatio-Spectral Power

**Figure 1:**
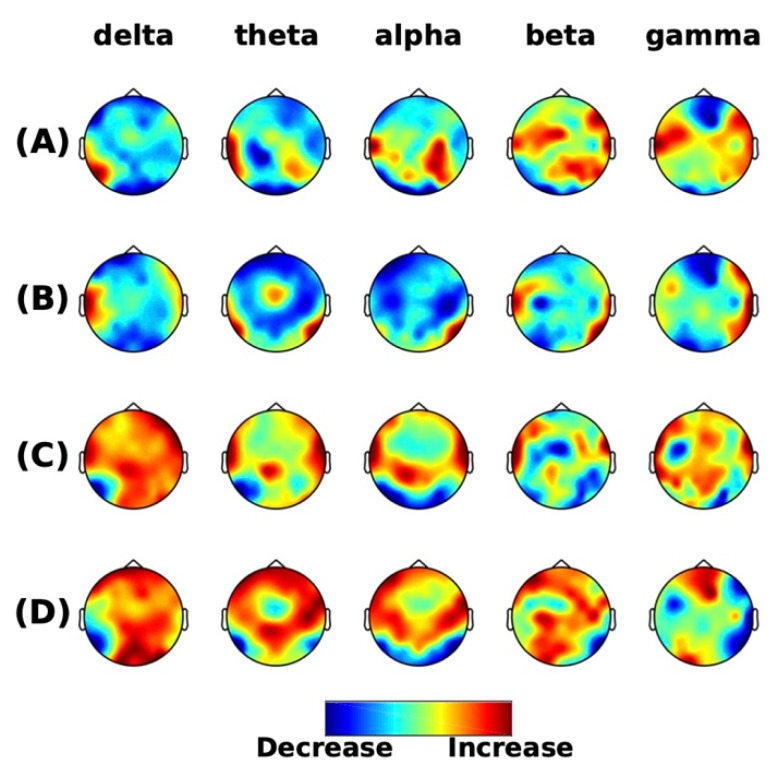
Resting-state frequency-domain analysis. Scalp topographies of the difference of grand averaged power from (**A**) the pre-intervention sessions (chiropractic-control), (**B**) post- and pre-control sessions, (**C**) post- and pre-chiropractic sessions, and (**D**) between interventions ((**C**) minus (**B**)). The cluster-based permutation tests showed non-significant differences in comparisons in (**A**–**C**), however, the absolute power in all frequency bands across the scalp was higher after the chiropractic intervention.

For all frequency bands, no differences in the power were seen between the pre-intervention sessions (no clusters) (Figure 1A), post- and pre-control intervention (1 positive cluster *p* = 0.055) (Figure 1B), and post- and pre-chiropractic spinal interventions (no clusters) (Figure 1C). There was a trend towards higher grand-averaged absolute power in all frequency bands after the chiropractic intervention compared to post-control intervention, which was similar or lower than the pre-control intervention. The difference between the two is shown in Figure 1D.

#### 3.1.2. DAR

The ANOVA revealed that there was no interaction between the interventions and sessions over DAR values (all *p* > 0.05). Descriptive statistics are given in Table 2.

#### 3.1.3. BSI

No interaction between interventions and sessions was found in any of the frequency band’s BSI values and directions (all *p* > 0.05). Descriptive statistics are given in Table 2.

#### 3.1.4. Source Localization

For all frequency bands, the sLORETA analysis showed that the pre-sessions were similar. A slight increase in activity was seen after the control intervention, however, neither of the interventions had any significant effect on brain activity (all *p* > 0.05).

### 3.2. SEPs

The number of epochs per subject used for the analysis were 799 ± 124 ([min, max] = [437, 956]).

#### 3.2.1. SEP Peaks

**Figure 2:**
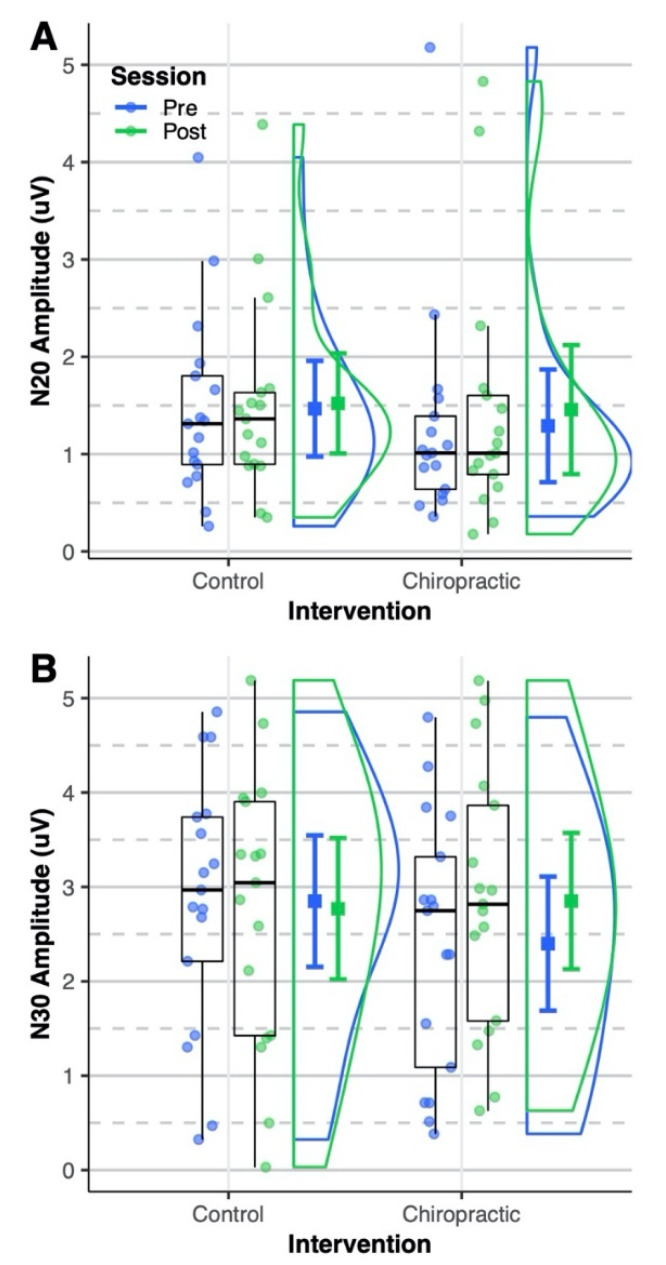
Somatosensory evoked potentials (SEPs) amplitude. Dots represent (**A**) N20 and (**B**) N30 amplitudes from all sessions of the analyzed subjects. Boxplots show the median, 25th and 75th percentiles. The error bars represent mean ± 95% CI. The distribution plots show the density distribution estimated by a Gaussian kernel with SD of 1.5. (**A**) The N20 amplitude was not affected by either intervention. (**B**) The N30 amplitude was similar for the pre-intervention sessions. After the chiropractic spinal manipulation, the N30 amplitude increased significantly, however, it was not changed after the control intervention. The figure was made using the code provided by Allen et al. [61].

**Figure 3:**
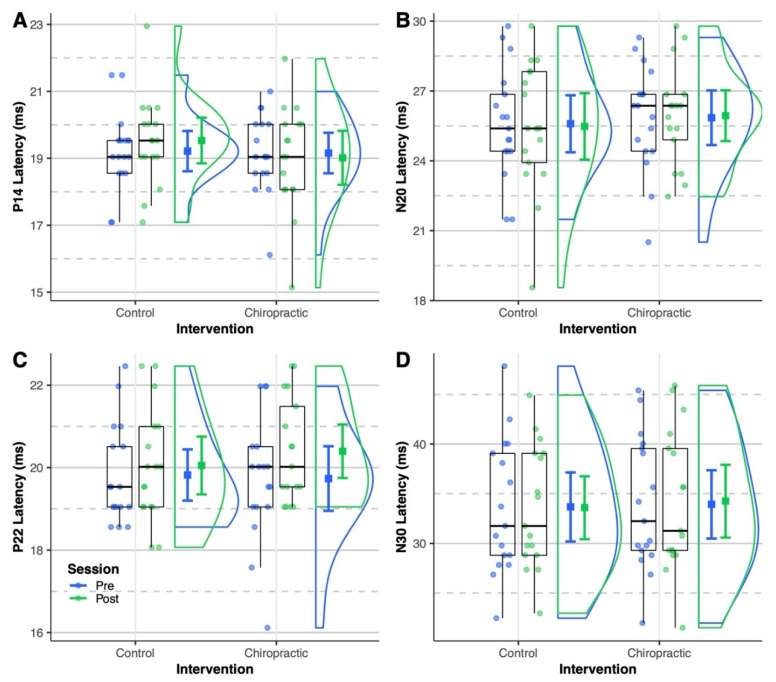
SEPs latency. Dots represent (**A**) P14, (**B**) N20, (**C**) P22, and (**D**) N30 latencies from all sessions of the analyzed subjects. Boxplots show the median, 25th and 75th percentiles. The error bars represent mean ± 95% CI. The distribution plots show the density distribution estimated by a Gaussian kernel with SD of 1.5. None of the SEPs latencies were significantly affected by either intervention.

The N20 amplitude was not affected by either intervention (all *p* > 0.05) (Figure 2A). The ANOVA (Table 3) showed a significant interaction of the intervention and session for the N30 SEP peak amplitude (*p* = 0.03). The post hoc analysis showed that the N30 amplitude was increased by 39% after the chiropractic spinal manipulation (*p* = 0.007; 95% CI = [0.11, 0.78]). The N30 amplitude was similar for the pre-intervention sessions (control vs chiropractic: *p* = 0.12; 95% CI = [−0.09, 0.99]) and there was no change after the control intervention (post vs pre: *p* = 0.95; 95% CI = [−0.56, 0.39]). Figure 2B shows the distribution of N30 amplitude across the four sessions. None of the SEPs latencies were significantly changed following either intervention (all *p* > 0.05) (Figure 3).

#### 3.2.2. Source Localization

The analyses were performed on 16 subjects because for one subject one of their sessions had an RV that was greater than 10%. The LORETA solution revealed five distinct areas during the 20–60 ms post-stimulus period; contralateral primary somatosensory cortex (SI), prefrontal cortex, cingulate, and bilateral secondary somatosensory cortices (SII). Therefore, a 5-source solution was assumed, and the dipoles were placed in these areas.

**Figure 4:**
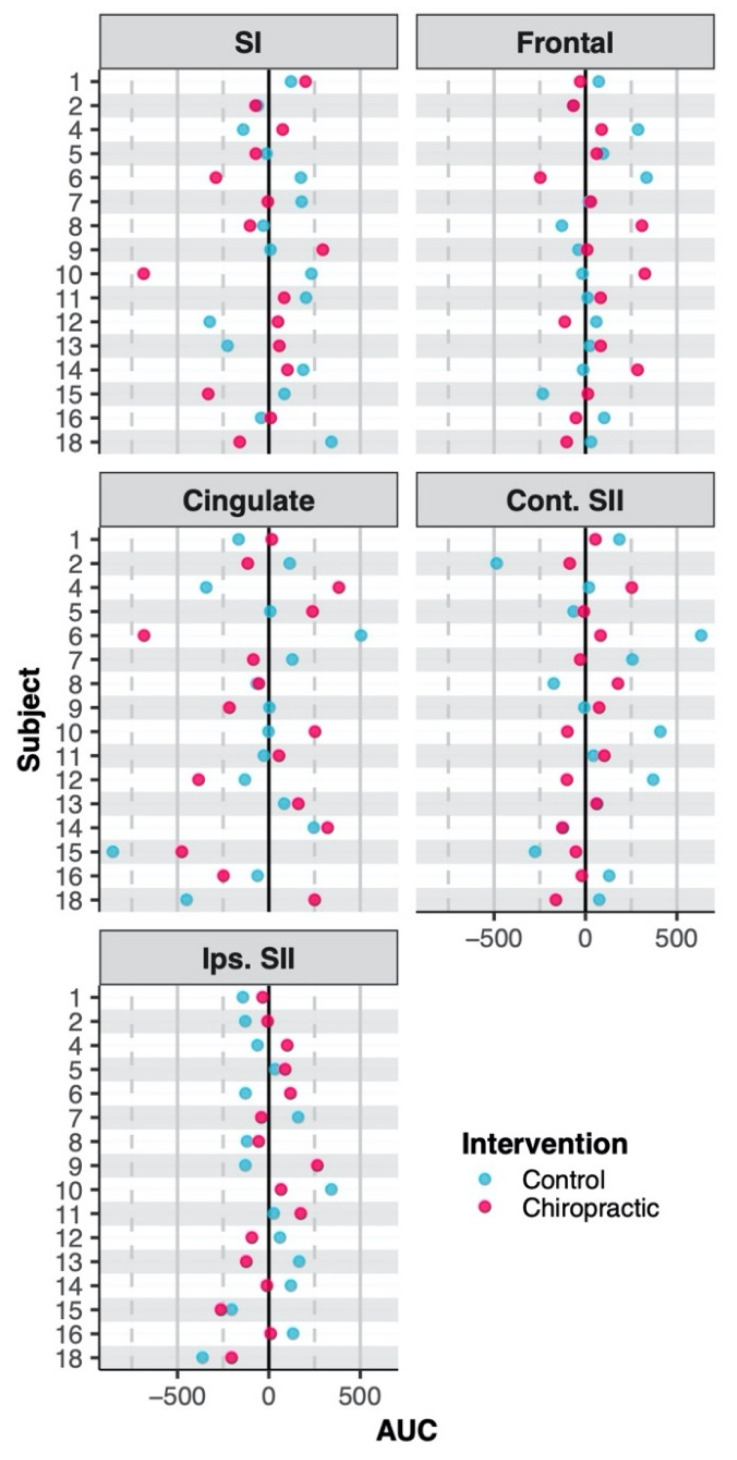
Area under the curve. The dots represent adjusted area under curve (AUC) with respect to the baselines set at 0 (black vertical line) (i.e. post-AUC minus pre-AUC). There were no effects on the strength of brain sources by either intervention. Abbreviations are same as in Table 4.

The ANOVA showed no interactions between the interventions and sessions for source strengths (all *p* > 0.05). Table 4 shows the coordinates of the brain sources and AUCs of their activity waveforms. The AUCs obtained are shown in Figure 4.

## 4. Discussion

In this randomized cross-over study, we evaluated the effects of a single session of chiropractic spinal manipulation versus a control intervention on stroke patients by investigating the change in the resting-state EEG, early somatosensory evoked potentials and their underlying brain sources. We found that chiropractic spinal manipulation increased the N30 amplitude of the somatosensory potential evoked by median nerve stimulation, but there were no changes in the N20 amplitude and latencies. There were no effects on the strength of the N30 peak brain sources by either intervention. There were no changes in the power of frequency bands, resting-state EEG source localization, delta-alpha ratio, and brain-symmetry index after either intervention. To the best of our knowledge, the current randomized study is the first of its kind to investigate these parameters in a stroke population following chiropractic spinal manipulation.

### 4.1. Resting-State

The current study showed a trend towards higher grand-averaged absolute power in all frequency bands after the chiropractic intervention compared to post-control intervention, which was similar or lower than the pre-control intervention. However, none of these increases were significant. In our previous study, performed on sub-clinical pain patients [37], we found no differences in the spectral power and in the sLORETA based source localization after the chiropractic spinal manipulation and control interventions. Similarly, in this study, neither of the interventions had a significant effect on the resting-state EEG, although there was a trend towards higher grand-average absolute power in all frequency bands. The probable reason for this is likely the non-uniformity in the type and location of the stroke in the subjects. This variation among subjects makes finding a statistical difference more difficult and may have required a larger sample size. Hence, there is a need for more research to explore longer chiropractic intervention periods (>1 session) and larger sample sizes or more homogenous subject groups in future studies of this kind. It is possible that this study was underpowered to show any potential changes following the chiropractic intervention. It is also possible, of course, that chiropractic spinal manipulation does not affect the resting-state spectral power.

Since DAR has been shown to be a predictive parameter of recovery in (sub-) acute stroke [21,62], it was used in this study to see if it was affected by the chiropractic spinal manipulation. It was found that DAR was not changed significantly with either the chiropractic spinal manipulation or control intervention. The DAR has been found to be increased in (sub-) acute stroke survivors compared to healthy persons [23,24]. However, it has been shown to be similar between chronic stroke survivors and healthy individuals [17]. Therefore, in this study of chronic stroke survivors, it is plausible that this parameter is not predictive of recovery in stroke, and therefore, it was not affected by the interventions.

Previously, in a study conducted in chronic stroke patients [17], BSI was found to be increased in the delta and theta bands but was unchanged in the alpha and beta bands. The same study also showed, using directional BSI, that the affected hemisphere had more resting-state power compared to the unaffected hemisphere, particularly in the delta and theta bands. In the present study, however, the BSI was not affected by the interventions. The possible reasons for this were inter-individual differences, the cross-over design of the study with less time between interventions, or more sessions of the treatment is required to affect the BSI. Since BSI is based on spectral power, it is also possible that chiropractic spinal manipulation does not affect the spectral power, and hence, does not affect BSI.

### 4.2. SEPs

In the current study, a 39% increase in the N30 SEP peak amplitude was observed following the chiropractic intervention. Previously, SEP studies looking at the effects of chiropractic spinal manipulation, compared to a control intervention, have shown that the N30 SEP amplitude decreased by 17–30% [32,35,36,39]. Lelic et al. [39] showed that this decrease in N30 SEP peak amplitude occurred in the prefrontal cortex, where there was a decrease of approximately 20% in the activity. Most of these studies were conducted in subclinical pain patients.

The N30 SEP peak is believed to be generated at the motor, premotor, and prefrontal cortices [63,64,65], and reflects sensorimotor integration [66]. Effective sensorimotor integration is required for learning new motor skills as well as for recovering from injuries such as stroke [67,68]. The prefrontal cortex, in particular, is of interest since it is associated with cognitive thinking and decision-making using the somatosensory input and information from other internal and external sources [69]. Early SEP peak amplitudes are known to be severely diminished or even absent in stroke populations [7,10,11,13,14]. It is, therefore, interesting that the chiropractic intervention showed an increase in the N30 SEP peak amplitude, considering previous studies in subclinical subjects have consistently reported a decrease in N30 SEP peak amplitude after chiropractic spinal manipulation [32,36,39,40]. The increase observed in the present study may reflect an improvement in early sensorimotor function that is related to the observed increase in strength found in a recent study of chronic stroke survivors after a single session of chiropractic spinal manipulation [41]. In that study [41], Holt et al. investigated the effect of chiropractic spinal manipulation on motor recovery in a chronic stroke population and found that, on average, plantarflexion muscle strength increased by approximately 64% after a single session of chiropractic care.

The source strength of the N30 SEP peak was not affected by the interventions. The possible reason for no significant changes in the source strength of the N30 SEP peak can be that the BESA analysis was performed on a head model of the healthy brain which did not cater for the number, location, and size of the lesion(s) of the participants in this current study. Using individual MRI to obtain a realistic head model of each subject could have improved the accuracy of source modeling, as suggested by previous simulation studies [70,71,72]. Therefore, this limitation should be kept in mind when interpreting the results.

## 5. Study Considerations

There are several reasons that no changes in the brain activity were seen in other parameters apart from the N30 amplitude after the chiropractic spinal manipulation intervention. These include that chiropractic spinal manipulation does not alter these parameters, or it may be because there are differences in the brain morphology of the stroke patients and non-uniformity of the type of stroke and affected brain regions. The enrollment criteria of future studies could be modified to include more homogenous patients with respect to stroke type and location. This would, of course, make the recruitment of patients more difficult, and the results of that study may not be generalizable to the majority of the stroke population.

This study was an exploratory study with a sample size of 19. Previous studies based on stroke patients had sample sizes similar to this study (10 to 21 participants) [17,20,25,62,73]. However, we recognize that the sample size used may not be large enough, and this study may, therefore, be underpowered to detect changes following spinal manipulation that occur in this population. Future studies that further explore the potential changes following chiropractic care for this population should consider increasing the sample size.

It is also important to keep in mind that previous work has shown that some neuroplastic effects of chiropractic spinal manipulation can, in some instances, remain for at least a week [74]. This may have affected the current study because it could have reduced or nullified the changes in this study, where both interventions were performed within a week. Future studies should control for this factor by having more than a week between the interventions, or they should use a parallel-group design.

## 6. Conclusions

The findings of the study suggest that in chronic stroke survivors, a single session of chiropractic spinal manipulation increases the cortical activity related to the somatosensory evoked N30 potential, which may alter the early sensorimotor functionality. Future studies should investigate the long-term effects of chiropractic care in this population group to understand better the impact of chiropractic care on cortical activity in stroke survivors.

## Figures and Tables

**Table 1:** Patients’ characteristics.

No.	Age (Years)	Type of Stroke	Area Involved	Affected Hemisphere	FM Score	Time Since Event (Months)
1	54	Ischemia	MCA	Left	55	24
2	51	Ischemia	ACA	Left	57	18
3	68	Hemorrhage	MCA	Left	41	60
4	75	Ischemia	ACA	Right	76	12
5	36	Ischemia	MCA	Left	64	18
6	61	Hemorrhage	MCA	Left	83	5
7	33	Ischemia	MCA	Left	55	5
8	48	Ischemia	MCA	Left	64	24
9	56	Ischemia	MCA	Left	63	5
10	58	Ischemia	MCA	Left	76	25
11	41	Hemorrhage	MCA	Right	64	20
12	62	Ischemia	MCA	Right	72	16
13	46	Ischemia	MCA	Right	54	13
14	33	Hemorrhage	MCA	Right	71	46
15	51	Ischemia	MCA	Right	46	23
16	66	Ischemia	MCA	Right	78	12
17 *	63	Hemorrhage	ACA	Right	21	50
18	58	Ischemia	MCA	Left	63	16
19 *	38	Hemorrhage	MCA	Right	58	3

*Note*. * = Patients excluded; MCA = Middle cerebral artery; ACA = Anterior cerebral artery; FM = Fugl-Meyer Score.

**Table 2:** DAR and BSI descriptive statistics.

Intervention	Session	DAR	BSI_1-25 Hz_		BSI_delta_		BSI_theta_		BSI_alpha_		BSI_beta_		BSI_gamma_	
			Value	dir	Value	dir	Value	dir	Value	dir	Value	dir	Value	dir
Control	Pre	2.37 ± 1.70	0.44 ± 0.17	0.08 ± 0.24	0.48 ± 0.17	0.11 ± 0.25	0.48 ± 0.15	0.11 ± 0.27	0.47 ± 0.21	0.03 ± 0.26	0.41 ± 0.16	0.01 ± 0.22	0.46 ± 0.10	0.07 ± 0.22
	Post	2.12 ± 1.55	0.42 ± 0.16	0.08 ± 0.23	0.47 ± 0.17	0.11 ± 0.24	0.48 ± 0.16	0.12 ± 0.27	0.44 ± 0.21	0.02 ± 0.25	0.39 ± 0.14	−0.01 ± 0.21	0.47 ± 0.14	0.04 ± 0.28
Chiropractic	Pre	2.48 ± 2.20	0.38 ± 0.11	0.11 ± 0.14	0.43 ± 0.15	0.15 ± 0.14	0.41 ± 0.14	0.15 ± 0.16	0.40 ± 0.17	0.04 ± 0.18	0.35 ± 0.08	−0.01 ± 0.14	0.40 ± 0.09	0.02 ± 0.21
	Post	2.05 ± 1.14	0.44 ± 0.21	0.09 ± 0.19	0.45 ± 0.18	0.11 ± 0.19	0.49 ± 0.24	0.11 ± 0.19	0.51 ± 0.25	0.06 ± 0.22	0.38 ± 0.21	0.01 ± 0.17	0.41 ± 0.16	0.03 ± 0.16

**Table 3:** ANOVA–N30 Amplitude.

Predictor	*df*_num	*df*_den	SS_num	SS_den	F	*p* Value
(Intercept)	1	16	501.38	112.10	71.56	0.00
intervention	1	16	0.58	7.61	1.21	0.30
session	1	16	0.57	2.29	3.99	0.06
intervention × session	1	16	1.20	3.37	5.71	0.03

*Note*. *df*_num indicates degrees of freedom numerator. *df*_den indicates degrees of freedom denominator. SS_num indicates sum of squares numerator. SS_den indicates sum of squares denominator.

**Table 4:** Coordinates (Talairach) and areas under curve (AUCs) of brain sources.

Region	Control					Chiropractic				
	X	Y	Z	AUC Pre	AUC Post	X	Y	Z	AUC Pre	AUC Post
SI	26 ± 11	−31 ± 9	38 ± 7	897 ± 492	941 ± 482	27 ± 6	−29 ± 11	36 ± 7	1001 ± 589	949 ± 473
Pre Frontal	3 ± 13	44 ± 3	16 ± 5	349 ± 208	383 ± 226	−4 ± 18	44 ± 6	11 ± 5	376 ± 276	418 ± 260
Cingulate	−22 ± 27	−5 ± 10	−6 ± 4	537 ± 354	474 ± 383	−18 ± 31	−4 ± 11	−6 ± 7	530 ± 412	494 ± 281
Cont. SII	27 ± 8	−52 ± 9	−14 ± 3	449 ± 236	515 ± 314	27 ± 12	−49 ± 13	−14 ± 2	511 ± 328	518 ± 286
Ips. SII	−28 ± 10	−52 ± 13	−9 ± 12	362 ± 172	348 ± 172	−28 ± 8	−48 ± 15	−12 ± 11	336 ± 191	336 ± 172

*Note*. Abbreviations: AUC = Area under curve; SI = Primary somatosensory cortex; Cont. SII = Contralateral secondary somatosensory cortex; Ips. SII = Ipsilateral secondary somatosensory cortex.
